# Eligibility for interventions, co-occurrence and risk factors for unhealthy behaviours in patients consulting for routine primary care: results from the Pre-Empt study

**DOI:** 10.1186/s12875-015-0359-x

**Published:** 2015-10-09

**Authors:** Elizabeth Randell, Timothy Pickles, Sharon A. Simpson, Clio Spanou, Jim McCambridge, Kerenza Hood, Christopher C. Butler

**Affiliations:** South East Wales Trials Unit, Centre for Trials Research, Cardiff University, 7th floor Neuadd Meirionnydd, Heath Park, Cardiff, CF14 4YS UK; MRC/CSO SPHSU, 200 Renfield Street, Glasgow, G2 3QB UK; School of Psychology, Faculty of Health & Social Sciences, University of Bedfordshire, Park Square, Luton, LU1 3JU UK; Department of Health Sciences, Seebohm Rowntree Building University of York, Heslington, York, YO10 5DD UK; Nuffield Department of Primary Care Health Sciences, University of Oxford, New Radcliffe House, Radcliffe Observatory Quarter, Woodstock Road, Oxford, OX2 6NW UK

**Keywords:** Screening, Health behaviours, Primary care, Physical activity, Diet, Smoking

## Abstract

**Background:**

Smoking, excessive drinking, lack of exercise and a poor diet remain key causes of premature morbidity and mortality globally, yet it is not clear what proportion of patients attending for routine primary care are eligible for interventions about these behaviours, the extent to which they co-occur within individuals, and which individuals are at greatest risk for multiple unhealthy behaviours. The aim of the trial was to examine ‘intervention eligibility’ and co-occurrence of the ‘big four’ risky health behaviours – lack of exercise, smoking, an unhealthy diet and excessive drinking – in a primary care population.

**Methods:**

Data were collected from adult patients consulting routinely in general practice across South Wales as part of the Pre-Empt study; a cluster randomised controlled trial.

After giving consent, participants completed screening instruments, which included the following to assess eligibility for an intervention based on set thresholds: AUDIT-C (for alcohol), HSI (for smoking), IPAQ (for exercise) and a subset of DINE (for diet). The intervention following screening was based on which combination of risky behaviours the patient had. Descriptive statistics, χ2 tests for association and ordinal regressions were undertaken.

**Results:**

Two thousand sixty seven patients were screened: mean age of 48.6 years, 61.9 % female and 42.8 % in a managerial or professional occupation. In terms of numbers of risky behaviours screened eligible for, two was the most common (43.6 %), with diet and exercise (27.2 %) being the most common combination. Insufficient exercise was the most common single risky behaviour (12.0 %). 21.8 % of patients would have been eligible for an intervention for three behaviours and 5.9 % for all four behaviours. Just 4.5 % of patients did not identify any risky behaviours. Women, older age groups and those in managerial or professional occupations were more likely to exhibit all four risky behaviours.

**Conclusion:**

Very few patients consulting for routine primary care screen ineligible for interventions about common unhealthy behaviours, and most engage in more than one of the major common unhealthy behaviours. Clinicians should be particularly alert to opportunities to engaging younger, non professional men and those with multi-morbidity about risky health behaviour.

**Trial registration:**

ISRCTN22495456

## Background

‘Risky’ health behaviours such as smoking, excessive drinking, lack of exercise and a poor diet remain key causes of premature morbidity and mortality globally [1]. The recent Global Burden of Disease study highlights that cancer and heart disease have now become the dominant causes of death and disability worldwide and that non-communicable diseases are increasing among adults [2]. The World Health Organisation (WHO) report that 61 % of cardiovascular deaths and over three quarters of the world’s ischaemic heart disease (the leading cause of death) can be accounted for by just 8 risk factors – alcohol use, tobacco use, high blood pressure, high body mass index, high cholesterol, high blood glucose, low fruit and vegetable intake, and physical inactivity [2]. These behaviours could be attributed to an unhealthy lifestyle the consequences of which account for roughly 70 % of health care expenditure [3]. The terminology used in current literature for these behaviours varies but for the purposes of this paper, the phrases ‘risky health behaviours’ and ‘unhealthy behaviours’ will be used alike.

In 2014 the Welsh Health Survey reported that 21 % of adults smoked, 42 % of adults admitted to drinking above the guidelines on at least one day in the past week, including 26 % who reported drinking more than twice the daily guidelines (sometimes termed binge drinking) and just 29 % met the guidelines for physical activity [4]. Additionally 58 % of adults were classed as overweight or obese. A key issue is that these unhealthy behaviours are known to often co-occur [5], for example, smokers typically have poorer diets, are less physically active, and consume more alcohol than non-smokers [6–8]. This increases not only the risk of disease [9] but is also a challenge for those trying to intervene [10]. In addition, health behaviours have also been shown to cluster in populations [11] with men, younger age groups and people of low socioeconomic status and education being more likely to engage in multiple risky health behaviours [12–15].

General practice in the U.K. can offer an ideal opportunity to identify beneficial lifestyle changes as over 80 % of patients attend consultations annually [16]. Patients expect practitioners to discuss health behaviours and be a reliable source of advice [17]. Given that patients attend an average of 5.5 consultations per year [18], GPs and practice nurses have the unique opportunity of being able to intervene across a range of behaviours in the same individual over time. However, opportunity is only part of the equation. With busy schedules, clinicians need to know how to use these opportunities to best effect. Barriers to achieving this lie in identifying who might most benefit from intervention (screening) and having the necessary skills and confidence to tackle these issues (intervention) [19].

Feedback from primary care practitioners highlights the need for more explicit guidance regarding screening and intervention for health behaviours and especially better methods of recognising multiple behavioural risks [20]. Routine screening for risky lifestyle behaviours in all patients may not be feasible, so targeting high risk groups – such as those with relevant health conditions such as hypertension, diabetes or particular populations [21] – may effectively assess and highlight those who would benefit from early intervention or referral to other services before more serious chronic conditions emerge [2].

Highlighting the risks associated with an unhealthy lifestyle is an important way of pre-empting health needs before they arise, equipping patients with resources that preserve future health and thus reducing possible disease risks [22]. This way of improving population health, although only part of an overall strategy, has been associated with substantial health benefits [22, 23]. Screening patients for risky health behaviours is therefore an important step in anticipatory care. Instead of reacting to the presentation of single risky health behaviours or problems resulting from them, anticipatory care suggests that if it is known that other risky behaviours are likely to follow, they could be addressed at the same time. Recent work by The King’s Fund [16] also confirms the need for a ‘holistic approach to policy and practice’ and shifting focus to tackling multiple health behaviours at once rather than single ones.

The aim of this study is to use data from the Pre-Empt trial to examine the numbers of patients consulting for routine care who would be eligible for an intervention about unhealthy behaviours according to current recommendations, and the co-occurrence of the big four risky health behaviours – lack of exercise, smoking, an unhealthy diet and excessive drinking – in a primary care population in order to help inform best practice in screening and identification of patterns of health behaviours.

## Method

The study was approved by the Multi-centre Research ethics Committee (MREC, 07/MRE09/11) and all Local Health Boards (LHBs) in Wales.

### Study design

Data were collected as part of the Pre-Empt study; a cluster randomised controlled trial with randomisation at the General Practice level [24].

### Participants

A total of 2067 patients who consulted primary care clinicians at 27 general practices across South Wales were screened to take part in the Pre-empt trial by completing a baseline questionnaire. Practices were a mixture of rural and urban with varying list sizes. Participants were consulting for a wide range of acute and chronic concerns, were aged 18 years or over and able to provide informed consent. The inclusion criteria to be eligible for the Pre-Empt trial was to screen above/below set cut points for at least one of the four risky health behaviours. There were no specific exclusion criteria, other than that the patient had to be able to understand and comply with the requirements as laid out in the study information sheet which meant that those who were unable to complete the questionnaires in English were excluded. In addition, after a brief discussion, the clinician (in both intervention and control practices) and the patient might decide that it would not be beneficial for the patient to participate in the trial.

### Procedure

Patient recruitment took place in practices in two intensive, one-week periods. During the recruitment week, patients entering the practice to see a clinician taking part in the study that day were given a flyer by the practice receptionist after booking in for their appointment. The flyer contained brief information about the study and invited the patient to speak with the researchers. If they were then happy to talk gain further information, the researchers then gave the patient a more detailed information sheet, discussed the study, answered any questions and took consent. Patients were then asked to self-complete a short baseline questionnaire which included screening instruments for assessing smoking [25], risky drinking [26], physical activity [27], and dietary behaviour [28, 29].

The screening instruments contained in the baseline questionnaire included;

- A subset of the Dietary Instrument for Nutrition Evaluation (DINE) measuring average fat and fibre intake along with a two item fruit and vegetable question [28, 29]. Patients screened positive for diet if they did not consume at least five portions of fruit and vegetables a day or ate three or more portions of high fat foods per week;
- The three item Alcohol Use Disorders Identification Test – Consumption Scale (AUDIT-C) [26]. This is a 3 item alcohol screen which asks about number and frequency of drinks consumed. A score of more than 3 for women or more than 4 for men screened positive;
- The Heaviness of Smoking Index (HSI) [25]. This 2 item screen asks how soon after waking they have their first cigarette and how many cigarettes an individual smokes per day. Any patient who smoked screened positive;
- The International Physical Activity Questionnaire (IPAQ short form) [27]. This asks about levels of physical activity in a ‘usual week’. If patients reported doing less than the recommended 30 min of vigorous or moderate physical activity five times a week screened positive for exercise.

The screening cut points were chosen specifically for the Pre-Empt trial and reflected threshold levels for each of the health behaviours which could trigger intervention in UK general practice. After each patient had completed the questionnaire, the researchers scored the measures and gave each patient a summary sheet identifying which risky behaviour they exceeded the threshold for to take pass to their GP in their appointment. As far as possible, patients were encouraged to complete any questions missed.

### Statistics

Descriptive statistics, χ^2^ tests for association and ordinal regressions were undertaken in IBM SPSS Statistics 20. Ordinal regressions (using a Cauchit link function due to underlying normality of the latent variable) were undertaken with number of risky behaviours individuals screened eligible for as the dependent variable (i.e. 0, 1, 2, 3 or 4 risky behaviours), and the following used as predictors:

1. Age;
2. Gender;
3. Marital status (as dichotomous Married/cohabiting vs. the rest variable);
4. Socio-economic classification (as dichotomous Managerial/Professional Occupation vs. the rest variable);
5. Number of Health Conditions [0 to 11]) based on asking patients about heart disease, diabetes, depression, stroke, arthritis, hypertension, high cholesterol, asthma, COPD, backache.

Tests of parallel lines were undertaken to check the proportionality assumption of these models and all were found to be valid.

The significance level was set at α = 0.05.

## Results

### Demographics

A total of 2802 patients were approached to take part in the study of which 2067 (73.8 %) consented and completed a baseline questionnaire. There were missing data for 1 participant.

Mean (SD) age was 48.6 (40.48) years for women and 54.9 (28.17) years for men [Table 1], with 787 (38.1 %) males and 1279 (61.9 %) females (and one gender not recorded, excluded). The majority of patients were married or cohabiting (63.3 %) and had managerial or professional occupations (42.8 %). The most common self-reported health conditions were ‘other’ (29.2 %), ‘backache’ (28.7 %) and ‘arthritis’ (23.8 %).

**Table 1:** Demographics and Socio-economic classification of participants

	Male (*N* = 787)		Female (*N* = 1279)		Overall (*N* = 2066)
Demographics	N	Mean (SD^a^) or %	N	Mean (SD^a^) or %	Mean (SD^a^) or %
Age (years)	674	54.9 (28.17)	1100	48.6 (40.48)	51.0 (49.90)
Marital Status (%’s based on 1781 responses):					
Single	116	17.1	222	20.1	19.0
Married/cohabiting	463	68.3	665	60.3	63.3
Divorced	54	8.0	103	9.3	8.8
Widowed	45	6.6	113	10.2	8.9
Socio-economic Classification^b^ (%’s based on 1781 responses):					
Managerial and Professional Occupations	231	37.9	428	46.0	42.8
Immediate Occupations	23	3.8	197	20.1	13.6
Small Employers and Own Account Workers	118	19.4	73	7.8	12.4
Lower Supervisory and Technical Occupations	105	17.2	63	6.8	10.9
Semi-routine and Routine Occupations	132	21.7	180	19.3	20.3
Self reported health condition (%’s based on 1540 responses)	N	%	N	%	%
Other	187	27.5	333	30.2	29.2
Backache	180	26.5	332	30.1	28.7
Arthritis	155	22.8	270	24.5	23.8
Hypertension	163	24.0	218	19.7	21.4
High Cholesterol	160	23.6	207	18.8	20.6
Depression	95	14.0	210	19.0	17.1
Asthma	94	13.8	174	15.8	15.0
Heart Disease	134	19.7	113	10.2	13.9
Diabetes	117	17.2	99	9.0	12.1
Stroke	37	5.4	39	3.5	4.3

^a^Standard Deviations inflated for clustering within practice

^b^As defined by the Office of National Statistics (http://www.ons.gov.uk/ons/guide-method/classifications/current-standard-classifications/soc2010/soc2010-volume-3-ns-sec--rebased-on-soc2010--user-manual/index.html)

There were two significant associations in reported health conditions between men and women, with men showing significantly higher rates of both heart disease (19.7 and 10.2 %, *p* < 0.001) and diabetes (17.2 and 9.0 %, *p* < 0.001).

### Screening: eligibility for interventions based on current recommendations

24.2 % of participants reported levels that would trigger intervention for one health behaviour only [Table 2]. The majority screened positive for two risky behaviours (43.6 %). Out of 2066 participants, only 4.5 % did not screen positive for any of the risk behaviours.

**Table 2:** Screening patterns for risky health behaviours

Behaviours	Male (*N* = 787)		Female (*N* = 1279)		Overall (*N* = 2066)
	N	%	N	%	%
No Behaviours	37	4.7	56	4.4	4.5
One health behaviour	197	25.0	303	23.7	24.2
Two health behaviours	331	42.0	570	44.7	43.6
Three health behaviours	177	22.5	273	21.4	21.8
All four health behaviours	45	5.7	77	6.0	5.9

Number and proportion of participants with one or more adverse health behaviours

Where patients screened positive for single health behaviours only [Table 3], the majority screened positive for low levels of exercise (12.0 % of all combinations) which was substantially more than those who screened positive for the next most common single health behaviour, diet (8.7 %). The numbers who screened positive for alcohol and smoking individually were substantially lower again at 2.8 and 0.7 % respectively.

**Table 3:** Screening patterns for single and co-occurring risky health behaviours

No. of behaviours	Behaviours	Male (*N* = 787)		Female (*N* = 1279)		Overall (*N* = 2066)
		N	%	N	%	%
0	No Behaviours	37	4.7	56	4.4	4.5
1	Exercise only	70	8.9	178	13.9	12.0
	Diet only	97	12.0	83	6.5	8.7
	Alcohol only	26	3.3	32	2.5	2.8
	Smoking only	4	0.5	10	0.8	0.7
2	Diet and Exercise	195	25.0	367	28.7	27.2
	Alcohol and Diet	79	10.0	56	4.4	6.5
	Alcohol and Exercise	22	2.8	80	6.3	4.9
	Diet and Smoking	20	2.5	32	2.5	2.5
	Alcohol and Smoking	9	1.1	11	0.9	1.0
	Exercise and Smoking	6	0.8	24	1.9	1.5
3	Alcohol, Diet and Exercise	96	12.0	143	11.2	11.6
	Diet, Exercise and Smoking	37	4.7	75	5.9	5.4
	Alcohol, Diet and Smoking	33	4.2	31	2.4	3.1
	Alcohol, Exercise and Smoking	11	1.4	24	1.9	1.7
4	Alcohol, Diet, Exercise & Smoking	45	5.7	77	6.0	5.9
	Alcohol	321	40.8	454	35.5	37.5
	Diet	602	76.5	864	67.6	70.9
	Exercise	482	61.2	968	75.7	70.1
	Smoking	165	21.0	284	22.2	21.7

Where behaviours co-occurred, diet and exercise were most common with 27.2 % screening positive for these behaviours. Thus 47.9 % did not attain recommended guidelines for diet and exercise either alone or combined – (diet only 8.7 %, exercise only 12.0 %, combined 27.2 %). Out of 16 possible combinations of health behaviours [Table 3], 5 combinations made up of 3 behaviours accounted for the majority screened positive for (66.0 %) – [Diet and Exercise], [Exercise only], [Alcohol, Diet and Exercise], [Diet only], [Alcohol and Diet]. Diet and exercise were most common being in 4 and 3 out of 5 of the groups respectively. There were significant associations with gender in 3 of these categories - Exercise only (males 8.9 %, females 13.9 %, *p* = 0.001), Diet only (males 12.0 %, females 6.5 %, *p* < 0.001), Alcohol and Diet (males 10.0 %, females 4.4 %, *p* < 0.001). There were also significant associations in Alcohol and Exercise (males 2.8 %, females 6.3 %, *p* < 0.001), and Alcohol, Diet and Smoking (males 4.2 %, females 2.4 %, *p* = 0.026). There was no significant association between genders when screening individually for Alcohol (males 3.3 %, females 2.5 %, *p* = 0.337) and Smoking (males 0.5 %, females 0.8 %, *p* = 0.586).

The results of the ordinal regressions [Table 4] indicated that:

**Table 4:** Ordinal regression results

Variable		Simple			Multilevel		
		Proportional odds ratio	95 % confidence interval	*p*-value	Proportional odds ratio	95 % confidence interval	*p*-value
Age		0.977	0.971–0.983	<0.001	0.977	0.971–0.983	<0.001
Gender	Male	1.270	1.045–1.543	0.016	1.270	1.042–1.548	0.018
	Female	(Reference)			(Reference)		
Marital Status	Single/divorced/widowed	1.084	0.891–1.319	0.422	1.084	0.890–1.320	0.424
	Married/cohabiting	(Reference)			(Reference)		
Occupation	All other occupations	1.269	1.047–1.528	0.015	1.269	1.044–1.543	0.017
	Managerial and professional occupations	(Reference)			(Reference)		
Number of health conditions (1–11)		1.050	0.984–1.120	0.139	1.050	0.982–1.122	0.152

Note that ‘4 risky behaviours’ is the reference category, so the proportional odds ratio relates to how much more likely the patient is to have either 0, 1, 2 or 3 risky behaviours in comparison to 4 risky behaviours

1. For a unit increase in age, the odds of 0, 1, 2 and 3 risky behaviours, in comparison to 4 risky behaviours, are 0.977 greater, i.e. for a unit increase in age, it is 2.3 % less likely to have 0, 1, 2 and 3 risky behaviours in comparison to 4 risky behaviours;
2. For Males, the odds of 0, 1, 2 and 3 risky behaviours, in comparison to 4 risky behaviours, are 1.270 greater than females, i.e. for males, it is 27.0 % more likely to have 0, 1, 2 and 3 risky behaviours in comparison to 4 risky behaviours than females;
3. Marital status is not predictive of number of risky behaviours;
4. For those in non-Managerial/Professional Occupations, the odds of 0, 1, 2 and 3 risky behaviours, in comparison to 4 risky behaviours, are 1.269 greater than those in Managerial/Professional Occupations, i.e. for those in non-Managerial/Professional Occupations, it is 26.9 % more likely to have 0, 1, 2 and 3 risky behaviours in comparison to 4 risky behaviours than those in Managerial/Professional Occupations;
5. Number of health conditions is not predictive of number of risky behaviours.

Multi-level and simple ordinal regressions produced similar results, meaning that there was minimal clustering in patterns of screening based on practice.

## Discussion

### Summary

Almost all adult individuals consulting for routine primary care screened eligible for an intervention about risky health related behaviours, and these behaviours tend to cluster together within individuals. The vast majority failed to achieve recommended threshold levels for both diet and exercise in particular. This study found a consistent socio-demographic gradient in the prevalence of multiple risk factors, with women, older age groups and those in managerial or professional occupations being more likely to exhibit all four risky health behaviours.

### Comparison with existing literature

This study found patterns in the prevalence of risk factors to be in line with previous research [12–15] therefore supporting evidence that screening in primary care should be targeted to efficiently address multiple as opposed to single health behaviours [30, 31] in those most at risk. Those with particular health conditions for example, cardiovascular disease or diabetes, may be more effectively assessed as potentially benefitting from intervention.

### Limitations

Consideration must be given to the practicality and impact of trying to implement screening and interventions. Due to the competing demands and time constraints experienced by clinicians in primary care [32, 33] coupled with a lack of confidence [30] and knowledge [34, 35] in being able to provide brief advice [36], routine screening may often be overlooked. As well, a lack of alternative services to refer patients to may deter clinicians from raising discussions about health behaviours in the first place.

A limitation of this study is its sample which was a subset of patients attending primary care who could speak English and who were willing to participate in a randomised controlled trial. In addition, there was a risk of selection bias as a result the relatively small sample which was confined to those attending a limited number of GP practices in South Wales. Another recognised weakness comes with the use of self-report measures for health behaviours. It is likely that more individuals are actually at risk than were identified as there is an evidenced tendency to over report levels of physical activity [37] and healthy eating [38] and under report levels of smoking [39].

### Implications for research and practice

In this study, screening was completed by the research team prior to patients going into their appointment. In presenting the clinician with a ready summary of exceeded thresholds, it was immediately clear which areas might be of benefit to discuss with the patient therefore relieving some of the pressure on their time [40]. This is obviously not reflective of everyday practice so looking at practical ways of utilising waiting time as well as capitalising on the roles of reception staff and nurses for implementing screening could have a significant impact on making it more achievable [41].

It is also worth reflecting on the use of the ‘gold standard’ screening tools – that is those deemed to be the benchmark by which outcomes are measured – currently being used in primary care and whether they are still the most appropriate in light of the evidence around co-occurrence. This study used a combination of these which are designed for single health behaviours. The study team were on hand to provide patients with these forms and be of assistance during the screening process if required, however this wouldn’t be viable for practice. The tools can be quite confusing and time consuming to complete. Evidence from focus groups in primary care show that shorter, easy to score screening instruments are more likely to be used with questions relating to general health and lifestyle being less stigmatizing and received better by patients [42]. Further research needs to focus on developing gold standard brief screening tools for multiple health behaviours.

Clinicians also need more guidance on how to then intervene with patients. Seale et al. [43] suggest that clinicians already providing advice on one health behaviour might actually find it easier to address a second at the same time. They need to be able to identify which behaviours are going to be most appropriate to target effectively with the potential for a kind of ‘spill-over’ from one intervention influencing a co-occuring unhealthy behaviour. Increasingly, research is showing that multiple unhealthy behaviours can be changed simultaneously though exactly how this might be tackled is still not clear [44]. What is apparent is that individuals who have already been successful in changing one risky health behaviour may be able to transfer that success to a second behaviour [45] though greater effects may be achieved when intervening for particular pairings of unhealthy behaviours such as physical activity and healthy eating [41]. The effectiveness of an intervention will not only depend on highlighting unhealthy behaviours but also patients’ readiness to change [40, 46].

## Conclusion

When undertaking screening in primary care, very few patients would screen ineligible for any intervention using current screening tools. Clinicians might reasonably expect to see risky health behaviours occur together in an individual, especially in pairs of behaviours with diet and exercise most commonly occurring. There are still many challenges in the detection of unhealthy behaviours in primary care and in the delivery of anticipatory care associated with them. Findings from this study add to current knowledge on screening patterns of risky health behaviours and multiple risk behaviours in primary care. The need to look at how to best identify those at risk as well as the practicalities of who is best placed to do this has also been identified. Instruments better suited to identifying multiple risky health behaviours need to be researched as well as ways in which clinicians can be supported to deliver screening. With more effective screening comes the need to establish effective interventions which are geared towards tackling multiple risky health behaviours.

## Abbreviations

AUDIT-C: Alcohol Use Disorders Identification Test Consumption
COPD: Chronic Obstructive Pulmonary Disease
DINE: Dietary Instrument for Nutrition Education
HSI: Heaviness of Smoking Index
IPAQ: International Physical Activity Questionnaire
WHO: World Health Organisation
